# Lipid-Induced Peroxidation in the Intestine Is Involved in Glucose Homeostasis Imbalance in Mice

**DOI:** 10.1371/journal.pone.0021184

**Published:** 2011-06-16

**Authors:** Matteo Serino, Aurélie Waget, Nicolas Marsollier, Myriam Masseboeuf, Gaëlle Payros, Catherine Kabani, Jessica Denom, Amélie Lacombe, Jean-Claude Thiers, Anne Negre-Salvayre, Serge Luquet, Rémy Burcelin, Céline Cruciani-Guglielmacci, Christophe Magnan

**Affiliations:** 1 Institut National de la Santé et de la Recherche Médicale (INSERM), U1048, Toulouse, France; 2 Université de Toulouse, UPS, Institut des Maladies Métaboliques et Cardiovasculaires (I2MC), Toulouse, France; 3 CNRS EAC 4413, Biologie Fonctionnelle et Adaptative, Paris, France; University College Dublin, Ireland

## Abstract

**Background:**

Daily variations in lipid concentrations in both gut lumen and blood are detected by specific sensors located in the gastrointestinal tract and in specialized central areas. Deregulation of the lipid sensors could be partly involved in the dysfunction of glucose homeostasis. The study aimed at comparing the effect of Medialipid (ML) overload on insulin secretion and sensitivity when administered either through the intestine or the carotid artery in mice.

**Methodology/Principal Findings:**

An indwelling intragastric or intracarotid catheter was installed in mice and ML or an isocaloric solution was infused over 24 hours. Glucose and insulin tolerance and vagus nerve activity were assessed. Some mice were treated daily for one week with the anti-lipid peroxidation agent aminoguanidine prior to the infusions and tests. The intestinal but not the intracarotid infusion of ML led to glucose and insulin intolerance when compared with controls. The intestinal ML overload induced lipid accumulation and increased lipid peroxidation as assessed by increased malondialdehyde production within both jejunum and duodenum. These effects were associated with the concomitant deregulation of vagus nerve. Administration of aminoguanidine protected against the effects of lipid overload and normalized glucose homeostasis and vagus nerve activity.

**Conclusions/Significance:**

Lipid overload within the intestine led to deregulation of gastrointestinal lipid sensing that in turn impaired glucose homeostasis through changes in autonomic nervous system activity.

## Introduction

It has now clearly been shown that nutrient sensing is a key factor in the regulation of energy homeostasis, especially that of glucose [1]. Indeed, daily variations in nutrient concentrations in both gut lumen and blood are detected by specific sensors located either in the gastrointestinal tract [2], [3] or in specialized central areas (mainly the hypothalamus or brainstem [4], [5]). In the case of the gastrointestinal tract, it has been known for many years that luminal nutrients stimulate the release of regulatory peptides from gut endocrine cells and also activate intrinsic and extrinsic neural pathways innervating the gut, in turn conveying signals to the brainstem through vagus afferent fibers [3], [6]. It has also been demonstrated that daily variations in nutrient concentrations in the blood can be directly detected by “nutrient sensitive neurons” (both glucose and fatty acid sensitive neurons located within the hypothalamus [7], [8]). Among nutrients, increasing evidence suggests an important role for an intestinal lipid-induced gut-brain neuronal axis to regulate energy homeostasis [9], [10] as well as direct hypothalamic fatty acid (FA) sensing [11], [12], [13]. With respect to glucose homeostasis, it has been shown that FA sensing contributes to nervous control of insulin secretion and action [14], [15].

Recent evidence suggests that alteration of these glucoregulatory pathways could be partly involved in the etiology of metabolic diseases such as obesity and/or type 2 diabetes. We have previously demonstrated that early changes in insulin secretion and action induced by a high-fat diet were related to a decreased sympathetic tone in rats [16]. The effect of the lipid-enriched diet were also observed when a triglyceride emulsion was directly infused into the 3^rd^ ventricle [17] or into the carotid artery [18], [19] without any change in plasma TG or fatty acid concentrations. In this latter experiment lipid infusion induced hepatic insulin-resistance and an increased glucose-induced insulin secretion in response to glucose tolerance tests, suggesting an adaptation of the endocrine pancreas to decreased insulin sensitivity [19]. In addition, the data emphasized that a high-fat diet may also affect sensing of dietary lipids by the gut [20]. The mechanisms involved in these deleterious processes could include inflammation and oxidative stress [21], [22]. Indeed, metabolic endotoxemia may contribute to the postprandial low-grade inflammatory state following ingestion of a high-fat meal [23] and finally contribute to the initiation of insulin-resistance and obesity [24]. These effects could be partly related to the accumulation of oxidant species like peroxidized lipids in the gut epithelial cells that could alter the mucosal metabolic pathways and enterocyte function [25].

The current study showed firstly that a 24 h lipid infusion impaired glucose-induced insulin secretion (GIIS) when administered through the intestine but not through the carotid artery. Secondly, we showed that the deleterious effects of intestinal lipid overload on glucose homeostasis could be prevented by administration of aminoguanidine (a nucleophilic hydralazine compound) which acts *in vivo* as an antioxidant agent against reactive oxygen species (ROS) and lipid peroxidation. Therefore, during the development of metabolic diseases, a lipid overload could impair energy homeostasis through mechanisms related to the early alteration of intestinal glucose sensing.

## Results

### A 24-hour intestinal but not brain lipid infusion impaired glucose homeostasis

**Figure 1:**
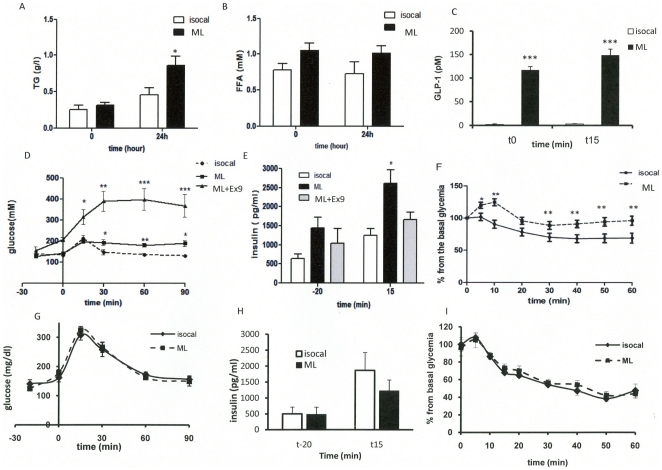
Effects of lipid infusion on glucose homeostasis in mice infused intragastrically for 24 hours with Medialipid (ML) or isocaloric solution (A, B, C, D, E, F) ; and in the brain (intracarotid) with ML or isocal (G, H, I). **A**: Plasma TG (g/l). **B**: FFA (mM). **C**: Plasma portal GLP1 concentration (pM) at the end of the intragastric perfusion (t0) and 15 min after glucose gavage. **D**: Time course of glycemia (mM) during the OGTT. **E**: Plasma insulin (pg/ml) 20 min before and 15 min after glucose challenge. **F**: Time course of glycemia during ITT. Results in 24 h intracarotid infused mice: **G**: Time course of glycemia (mM) during OGTT. **H**: Plasma insulin (pg/ml) 20 min before and 15 min after glucose. **I**: Time course of glycemia during ITT. (n = 8) *p<0.05, **p<0.01, ***p<0.001 compared to controls.

The intragastric ML infusion increased the plasma TG concentration when compared with the isocaloric infusion (Figure 1A) though conversely no change in plasma FFA concentrations was observed (Figure 1B). The 24 h intracarotid ML infusion did not increase either the plasma FFA or TG concentrations when compared with the controls (data not shown). Blood glucose and plasma insulin concentration remained unchanged after both types of infusions. The GLP-1 concentration in the portal vein was markedly increased by the intragastric ML perfusion (Figure 1C; t0). To determine the impact of the intestinal or intracarotid lipid overload on the control of glucose homeostasis we first investigated the time-course of glycemia in response to an oral glucose tolerance test (OGTT). At the end of the intragastric ML infusion, the blood glucose profile during OGTT was significantly higher in ML when compared with the isocal. In ML mice treated with the antagonist GLP1 receptor exendin 9 (Ex9) hyperglycemia during OGTT was much significantly higher than in ML mice (Figure 1D), thus suggesting that GLP1 signaling pathway is involved in glycemic response during OGTT, probably through modulation of insulin secretion. Indeed, in ML mice glucose intolerance was associated with an increased plasma insulin concentration 15 min after the glucose challenge. Such increased hyperinsulinemia was no more observed in ML mice treated with Ex9 (Figure 1E). In addition, insulin was less efficient in decreasing the glycemia in intragastric-ML infused mice (Figure 1F). In intracarotid-infused mice, ML did not change the glycemic (Figure 1G) or the insulinemic profiles (Figure 1H) during the OGTT when compared to controls. Following the intracarotid ML infusion, there was no change in the time-course of glycemia during ITT compared to controls (Figure 1I).

### Intragastric lipid infusion induced lipid droplet accumulation in jejunum but no inflammation

**Figure 2:**
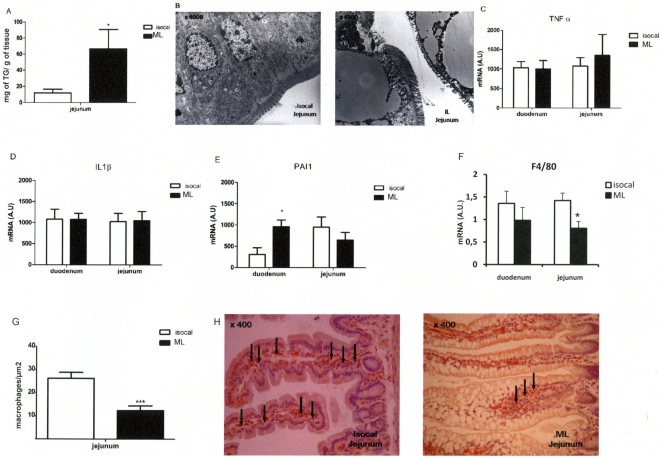
Intestinal Intestinal lipid content and markers of inflammation in isocaloric or ML intragastrically infused mice. **A**: TG content (mg/g of tissue) in jejunum. **B**: Electronic microscopy of jejunum (×4000). **C**,**D**,**E**, **F** : mRNA expression of TNFα (**C**), IL1β (**D**), PAI-1 (**E**) and F4/80 (F). **G**: Number of macrophages (F4/80), **H**: F4/80 immunohistochemistry. (n = 8) *p<0.05, **p<0.01, ***p<0.001 compared to controls.

The intestinal lipid content was increased in the jejunum (Figure 2A). Electron microscopy showed large lipid droplets in intestinal epithelial cells (Figure 2B). After the ML intestinal infusion the concentration of mRNA coding for the inflammatory cytokines, TNFα and IL1β was not increased when compared with mice infused with the isocaloric infusion (Figures 2C,D). However, the PAI-1 mRNA content was increased only in the duodenum (Figure 2E). In addition, the number of macrophages, as determined by F4/80 immunohistology (Figures 2F,G), was slightly decreased in the duodenum and was 2-fold less in the jejunum of intragastrically ML-infused mice when compared with the controls. In addition neither the IFNγ nor CD3 mRNA content, both markers of macrophages, changed in either group (data not shown).

### Intragastric lipid-infusion induced oxidative stress

**Figure 3:**
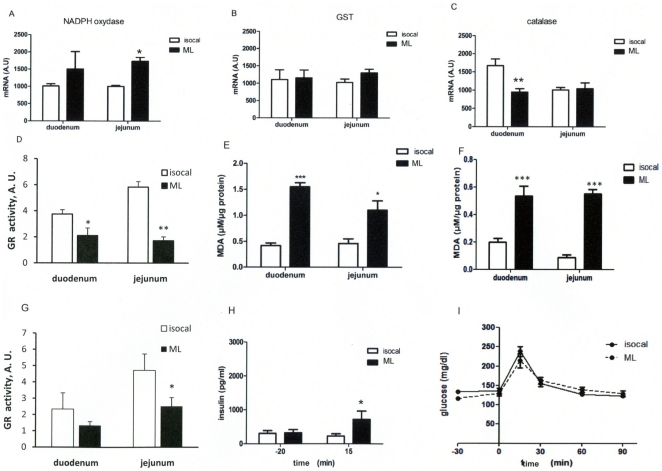
Markers of oxidative stress in intragastric-infused mice. **A**,**B**,**C**: mRNA expression of NADPH oxidase (**A**), GST (**B**), catalase (**C**). **D**: Activity of glutathione reductase in duodenum and jejunum. **E**: Lipid peroxidation by the expression of MDA (µM/µg protein). F, G, H, I: Infusion of isocaloric and Medialipid solution over 6 h. **F**: MDA production. **G**: Glutathione reductase activity in duodenum and jejunum. **H**: Plasma insulin (pg/ml) 20 min before and 15 min after glucose challenge. **I**: Time course of glycemia (mM) during OGTT. (n = 8) *p<0.05, **p<0.01, ***p<0.001 compared to controls.

The mRNA concentrations in the duodenum and jejunum of the genes coding for both oxidative (NADPH oxidase) (Figure 3A) and anti-oxidative (GST, catalase) enzymes (Figure B,C) were quantified. The data show statistical differences between ML and isocaloric infused mice (Figure 3A and 3C). Importantly, the activity of the anti-oxidative enzyme glutathione reductase was reduced in the duodenum and jejunum of ML-infused mice when compared to controls (Figure 3D). Furthermore, lipid peroxidation, as assessed by MDA production, was increased in both the 2 segments of ML-infused intestines (Figure 3E) when compared to controls. Importantly, in mice infused with ML for only 6 hours MDA production was already increased (Figure 3F) and the antioxidant activity, as witnessed by the glutathione reductase activity, was reduced (Figure 3G). This was accompanied by a state of hyperinsulinemia (Figure 3H) although at this early time point, glucose tolerance was unchanged (Figure 3I).

Interestingly, brain ML infusion did not increase MDA production suggesting that the duration of the infusion was not sufficient (data not shown).

### Aminoguanidine prevented the ML-induced glucose intolerance and insulin resistance

**Figure 4:**
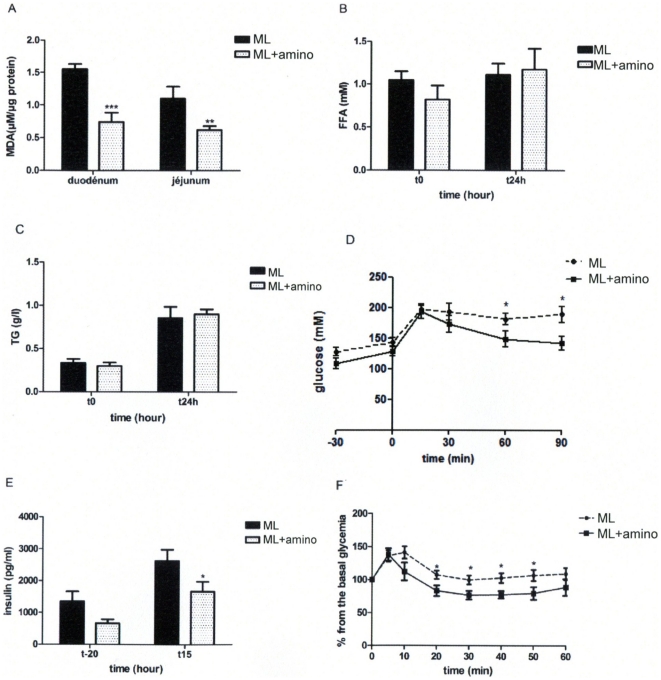
Effects of aminoguanidine on glucose homeostasis. Effect of aminoguanidine treatment on ML intragastrically infused mice. **A**: Malondialdehyde (MDA) production in duodenum and jejunum at the end of the infusion. **B**: FFA (mM). **C**: TG (g/l) concentration before (t0) and after (24 h) the perfusion. **D**: Time course of glycemia (mM) during OGTT and **E**: Plasma insulin (pg/ml) 20 min before and 15 min after glucose challenge. **F**: Time course of glycemia after insulin load. (n = 8) *p<0.05, when compared to ML.

To reverse the impact of ML on intestinal lipid peroxidation mice were administered with aminoguanidine for one week and then infused with ML for 24 hours. Following the 24 h-lipid infusion, MDA production was lower in the duodenum and jejunum from the aminoguanidine-treated mice (Figure 4A). The aminoguanidine treatment had no impact on mice infused with the isocaloric solution (data not shown) and did not modify plasma FFA (Figure 4B) and TG (Figure 4C) concentrations neither before nor after the ML infusion. The glycemic profile obtained in response to the oral glucose challenge was improved in aminoguanidine treated mice compared with control ML mice (Figure 4D). Moreover, aminoguanidine treatment decreased insulin secretion following glucose challenge (Figure 4E) and improved insulin tolerance (Figure 4F).

### Intragastric lipid-infusion and vagus nerve activity

**Figure 5:**
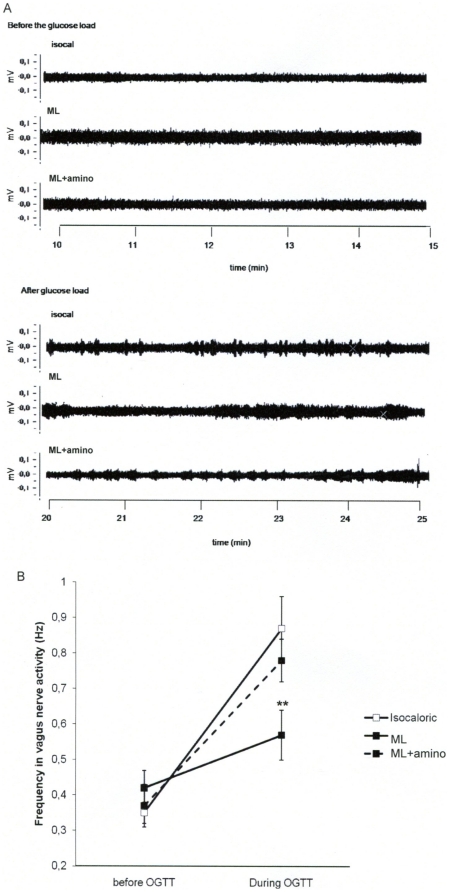
Activity of the vagal nerve. **A**: Recordings during 5 min before (upper panel) and 5 min after (lower panel) the glucose load in isocaloric (isocal), Medialipid (ML) or, aminoguanidine treated-ML (ML+ amino) conditions. **B**: Frequency of the vagal activity recorded before and during OGTT. (n = 8). **p<0.01 vs isocaloric.

The enteric glucose detection activates the “gut-brain axis” *via* the parasympathetic nervous system to transmit the glucose message to cerebral areas such as the brainstem. Under basal conditions, vagus nerve activity was similar in all groups (Figure 5A, upper panel and Figure 5B). In contrast, during the OGTT the sequences of nerve spikes were different between mice infused with ML or isocaloric solution (Figure 5A, bottom panel). In the control group fragments of recording displayed a type of response with clearer time intervals between events when compared with the ML-infused mice (Figure 5A, bottom panel) and the frequency of events was significantly higher (0.87±0.03 vs. 0.57±0.05 Hz, p<0.01, Figure 5B). The duration of the evoked events was also shorter in controls than in ML infused mice indicating changes in frequency of discharges (Figure 5A, bottom panel). Aminoguanidine treatment normalized vagus nerve activity in response to the OGTT (Fig. 5A, bottom panel: 0.87±0.03 vs. 0.78±0.06 Hz, NS, Figure 5B).

## Discussion

The present study showed that in mice a 24 h intragastric but not an intracarotid lipid overload, that mimicked the daily fat load during high-fat feeding, impaired both glucose and insulin intolerance and hence the overall glucose homeostasis. This was linked to an increased lipid content in the jejunum. Furthermore both duodenum and jejunum showed oxidative and peroxidized lipid induced stresses that preceded the impaired glucose homeostasis. The lipid effects were prevented by a prior aminoguanidine treatment. Our data suggest that a short-term lipid overload, which preferentially targets the intestine glucose sensor and gut-brain axis, is sufficient to initiate features of impaired glucose homeostasis. Mechanisms could involve excessive lipid peroxidation which can be a consequence of increased oxidative stress.

As mentioned above, the major consequence of intragastric lipid infusion was to deregulate glucose homeostasis including both glucose and insulin intolerance. It must be pointed out that in response to an oral glucose load the plasma insulin increased more in ML mice than in controls, suggesting an adaptation to glucose intolerance and decreased insulin sensitivity. However, this result could also be linked to the plasma GLP-1 concentration, which was significantly higher in ML-infused than in control mice. Both glucose and insulin intolerance observed in ML-intragastrically infused mice occurred without any change in plasma FFA concentrations. By contrast, intragastric ML infusion increased plasma TG concentrations compared to controls. This increased TG concentration was probably not responsible for the impaired glucose homeostasis, since aminoguanidine treatment, which had a preventive effect on the parameters tested, did not decrease plasma TG concentrations. In addition, both increased GLP-1 and insulin concentrations were not sufficient to counteract glucose intolerance. Thus we hypothesized that the change in glucose homeostasis could be related to a change in autonomic nervous system (ANS) activity. Indeed, it has been well-described that activation of the parasympathetic nervous system was necessary for normal meal-induced insulin secretion [26], [27]. Particularly, a study by Ahren and Holst showed that the pre-absorptive insulin response to meal ingestion in humans was largely attributed to autonomic activation [27]. The authors concluded that this cephalic insulin response was required for normal postprandial glucose tolerance, and that GLP-1 did not contribute to the pre-absorptive cephalic phase of the insulin response to a meal [27]. In our model, the deregulated parasympathetic nerve activity in response to oral glucose may, at least in part, explain the glucose intolerance despite the high GLP-1 concentration.

In our model, vagus nerve activity was similar in all groups under basal conditions and during the OGTT, there was no increase in vagus nerve activity in ML infused mice. Thus, intestinal ML infusion may lead to deregulation of afferent signals and finally overall vagus nerve activity that could in turn reduce the neural component of glucose homeostasis. Interestingly, it must be pointed out that the expression of plasminogen activator inhibitor-1 (PAI-1) was increased in the duodenum of ML-infused mice. Previous reports have shown that PAI-1 expression was upregulated in neurons after experimental peripheral nerve injury [28], [29]. Taken together, both the vagus nerve recordings and PAI-1 expression suggest deregulation of the enteric nervous system following ML infusion. How may ML infusion into the intestine lead to a change in vagus nerve activity? It has been demonstrated that intestinal infusions of oleate and glucose activated myenteric neurons in the duodenum and jejunum in the rat [30]. Such sensing could be deregulated in the presence of lipid overload and consecutive oxidation/inflammation processes. Functional changes in the enteric nervous system have been observed during inflammation [31], [32], [33]. However, it must be pointed out that inflammation was not significantly increased in our model. Indeed, neither IL1-β nor TNF-α expression were increased in the intestines of ML-infused mice. This could be related to the short time of the infusion (24 h) and we cannot exclude that a longer infusion period may induce inflammation and macrophage infiltration as described in other models [22], [25], [34]. In contrast, oxidative stress was activated in both duodenum and jejunum of ML-infused mice compared to controls, as indicated by the increased MDA production. Oxidative stress has been also shown to induce changes in enteric nervous system activity. For example, it has been demonstrated that the specific effects of the cytotoxic secondary lipid oxidation product, 4-hydroxynonenal (10^−8^–10^−4^ M) on intact sheets of rat jejunum was to stimulate chloride secretion mediated by prostaglandins and the enteric nervous system [35]. Oxidative stress is also associated with peripheral nerve dysfunction observed in diabetes. A study by Kellogg et al. demonstrated that oxidative stress was an important regulator of diabetic neuropathy [36]. In that study, COX-2 inactivation (using COX-2(−/−) diabetic mice) had a protective effect against the slowing of motor and sensory nerve conduction and impaired nerve antioxidative defense that were induced by diabetes. This protective effect was not seen in the wild-type (COX-2(+/+) diabetic mice [36]. Deregulated vagus nerve activation in ML-infused mice could also be related to the high GLP-1 level [37], [38]. Aminoguanidine has been previously demonstrated to have a protective effect on blood and tissue lipid peroxidation in jaundiced rats with endotoxemia induced by LPS [39]. Aminoguanidine is also a potential therapeutic agent for preventing the generation of advanced glycation end products (AGEs) in diabetes mellitus [40]. Our data showed that increased lipid peroxidation is induced by the ML infusion as early as after six hours, accompanied by an excessive insulin secretion. However, these features preceded glucose intolerance. Therefore, we suggest that the alteration in gut glucose sensing is an early mechanism within the time course of impaired glucose homeostasis.

In conclusion, our data show that a very short-term lipid stress impairs intestinal glucose sensing, through mechanisms that involve lipid peroxidation and probably oxidative stresses that target the gut-to-periphery neural axis. These biochemical events were prior to the later impairment of glucose homeostasis. Thus, gut could be a target organ for the development of new drugs aimed at regulating glucose homeostasis.

## Methods

### Animals and research design

#### Ethical statement

The following animal experimental procedures were approved by the local ethical committee of the Rangueil hospital and by the local ethical committee of the University of Paris Diderot (permit number: A75-13-17).

#### Animals housing

Eleven-week-old C57BL6/J (Charles River, L'Arbresle, France) male mice were housed in a controlled environment (inverted 12-h daylight cycle, lights off at 10:00 a.m.) with free access to food and water. All mice were fed with a normal carbohydrate diet (NC : proteins 22%, glucides 67%, lipids 11% of total kcal).

#### Research design

An indwelling catheter was installed in the stomach or in the carotid artery towards the brain. Following insertion of the catheters, mice were allowed to recover for one week post-surgery and to reach their pre-surgical body weight. On the day of experiment the catheters were connected to infusion systems that enabled the animal to remain in its cage. Mice were infused over 24 h with a triglyceride emulsion (Medialipid 20%; 18 Kcal/24 h, KabeVitrum, Stockholm, Sweden) or an isocaloric solution (Nutriflex lipid, B Braun, France). Medialipid contains 200 g/L of lipids (mainly soy oil) and Nutriflex is composed of 57 g/L of amino acids, 144 g/L of glucose and 40 g/L of lipids. It is noteworthy that this lipid infusion rate corresponds to the amount of lipid absorbed over 24 hours by mouse fed a high-fat diet [41]. A subset of mice was infused for six hours only, to evaluate the effect of a very short-term infusion on glucose homeostasis. At the completion of the infusions, different metabolic analyses were performed as described below. The duodenum, jejunum and the hypothalamus were collected and stored at −80°C. In another set of experiments, a group of mice had free access to drinking water complemented with a solution of aminoguanidine (100 mg/ml, Sigma Aldrich, Saint Louis, MO), during the week of recovery from surgery and before and during the intragastric or intracarotid 24-hour infusions.

### Surgical procedures and plasma parameters

#### Intragastric catheter

Mice were anesthetized with isoflurane (Abbott, Rungis, France), the hair shaved, and a 4-mm laparotomy performed under the thoracic cage, on the left side. The upper stomach was gently pierced with a needle and a catheter was inserted into the hole. It was secured by surgical glue (Histoacryl, 3M, Health Care, St. Paul, MN), and the other end of the catheter was tunnelled under the skin, exteriorized and closed at the back of the neck.

#### Intracarotid catheter

The long-term infusion technique under unrestrained conditions was used, as previously described [42]. Briefly, 7 days before the beginning of the infusion, mice were anesthetized with isoflurane (Abbott, Rungis, France) for the insertion of a catheter into the carotid artery towards the brain. Then, the catheter was exteriorized at the top of the head and attached to a swivelling infusion device, allowing the animal free access to food and water.

#### Plasma parameters

Before and after the 24 h-infusion, glycemia was measured and 20 µl of blood were collected from the tip of the caudal vein to analyse plasma fatty acid (FA) and triglyceride (TG) concentrations. The blood was immediately centrifuged and plasma frozen until assay.

### Tolerance tests

#### Oral glucose tolerance test (OGTT)

After the 24 h-infusion, mice were disconnected from the infusion system, allowing free moving in the cage. After twenty minutes, intragastric infused mice were gavaged with a glucose solution (2 g/kg). The glycemia was determined by a glucometer (Accu Chek, France) from 2 µl collected from the tip of the tail vein at times 0, 15, 30, 60, 90 and 120 min. In addition 20 µl of blood were sampled 20 min before, and at 15 and 60 min after the glucose gavage, in order to measure insulinemia. Blood was immediately centrifuged and plasma was frozen until insulin assay.

#### Insulin tolerance test (ITT)

A single dose of insulin was injected (0.05 U/ml, 10 µl/g, ip). The glycemia was measured in tail blood at times 0, 5, 10, 15, 20, 30, 40, 50 and 60 min.

#### GLP-1 sample collection

In mice allocated for assessment of portal vein GLP-1 concentrations, blood was collected in the presence of Diprotin A (Ile-pro-ile, 0.1 nM, Sigma-Aldrich, Saint Louis MO) and heparin at the end of the 24-hour infusion, and fifteen minutes following the glucose challenge. A rapid anesthesia was induced by an intraperitoneal injection of a mix of Ketamine 1000 (Vibrac, France) and Xylazine (Rompum 2%, Bayer health care, France, 100 and 10 mg/kg i.p., respectively) to obtain the portal vein samples.

#### Plasma parameters

Plasma insulin concentrations were determined in 10 µl using the mouse ultrasensitive insulin ELISA kit (Mercodia, Upsala, Sweden) and plasma GLP-1 concentrations were determined in 100 µl using the Glucagon-Like Peptide-1 (Active) ELISA kit (Linco Research). Plasma FFA concentration was assessed by the NEFA C kit (WAKO) using 8 µl. Plasma triglyceride concentration was determined in 3 µl using the triglycerides enzymatic PAP150 kit (Biomerieux).

### Biochemical assays

#### Protein extraction

Whole intestine and hypothalamus were solubilized by a RIPA solution, (Tris 1 M pH 7,5 (Sigma), Triton 10× (Sigma), NaCl 5 M, NaF 1 M (Sigma)) with antiprotease solution (aprotinin at 1.5 mg/ml (Euromedex), leupeptin at 1 mg/ml (Sigma), PMSF at 100 nM (Acros Organics, France) and sodium orto vanadate at 100 mM (Sigma)), respectively in 300 and 150 µl. After 30 min incubation, samples were centrifuged for 10 min at 1000 rpm, 4°C. The protein concentration was determined with the DC protein assay kit (Biorad, Marne la Coquette, France).

#### Lipid peroxidation

The reaction of malondialdehyde (MDA) with thiobarbituric acid (TBA) has been applied to assess lipid peroxidation in biological material. The reaction yields a red MDA-TBA adduct, the product of 2 mol of TBA plus 1 mol of MDA. The colored complex can be quantified spectrophotometrically from its visible absorbance (λ_max_ 532 nm). Briefly, 200 µl of duodenum and jejunum or 50 µl of hypothalamus protein extract was colored by the reaction with 0.5 M HCl, and TBA (Acros organics) during a dry bath- incubation (10 min, 95°C). After cooling, 2 ml of butanol (Sigma Aldrich) were added and the mixture gently shaken. After centrifugation (10 minutes at 12000 rpm, 4°C) the upper phase containing the colored lipid peroxide was collected and the absorbance was read at 532 nm by a spectrophotometer (WPA, Biowave).

#### Glutathione reductase

Glutathione (GS-SG) was reduced by glutathione reductase to oxidized glutathione (GSH) and NADP^+^. NADPH absorption (340 nm) was correlated with the glutathione reductase activity. On a 96-well plate, glutathione reductase was determined in the intestinal protein extract with a glutathione reductase assay kit (Sigma, Rabalot, France).

#### Triglyceride content

Total lipid was extract by the traditional Folch method from 50 mg of tissues mixed with 1 ml of Folch solution (chloroform/methanol: 2/1; v/v) (Sigma Aldrich, Saint Louis MO). The dried lipid extract was diluted with 500 µl of isopropanol (Sigma Aldrich, Saint Louis MO) to perform the triglyceride assay (triglycerides enzymatique PAP150; biomerieux)

#### mRNA expression

Total mRNA from duodenum and jejunum were extracted using TriPure reagent (Roche, Basel, Switzerland). The concentration of mRNAs was evaluated by quantitative RT-PCR analysis. The primers used were: TNFα (r: TTCGGAAAGCCCATTTGAGT, f: TGGGACAGTGACCTGGACTGT), IL-1β (r: CATCAGAGGCAAGGAGGAAAAC, f: TCGCTCAGGGTCACAAGAAA), PAI-1 (r: CCGAACCACAAAGAGAAAGGA, f: ACAGCCTTTGTCATCTCAGCC), F4/80 (r: GCAGGCGAGGAAAAGATAGTGT, f: TGACAACCAGACGGCTTGTG), CD3 (r: ATGCCCCAGAAAGTGTTCCA, f: TCCGCCATCTTGGTAGAGAGA), IFNγ (r:TGACTGTGCCGTGGCAGTA, f: TTGGCTTTGCAGCTCTTCCT), NADPH oxidase (r: TCGACACACAGGAATCAGGAT, f: GGTTGGGGCTGAACATTTTTC), GST (r: CCATCACTTCGTAACCTTGCC, f: AAGAATGGAGCCTATCCGGTG), catalase ( r: TCCGCTCTCTGTCAAAGTGTG, f: AGCGACCAGATGAAGCAGTG). PCRs were performed using an AbiPrism 7900 Sequence Detection System instrument and software (Applied Biosystems, Foster City, CA, USA). The concentration of each mRNA was normalized for RNA loading for each sample using RPL19 rRNA (r: CCTTGTCTGCCTTCAGCTTGT, f: GAAGGTCAAAGGGAATGTGTTCA) as an internal standard.

#### Immunohistology of macrophages, F4/80 staining

0.5 cm of both duodenum and jejunum were fixed in 4% paraformaldehyde (Sigma Aldrich). 8 µm transversal tissue sections were deparaffinized and rehydrated. Sections were blocked in normal goat serum and incubated overnight with primary rat anti-mouse F4/80 monoclonal antibody (1/1,000; Serotec, Oxford, U.K.). Endogenous horseradish peroxidase activity was quenched by incubation with 3% hydrogen peroxide for 20 min. Secondary antibody staining was performed using goat anti-rat biotinylated IgG Ab (1/1000, 30 min, room temperature) and streptavidin conjugated horseradish peroxidase (1/500, 30 min, room temperature) (Sigma Aldrich) and detected with 3,3-diaminobenzidine (Sigma Aldrich). Sections were counterstained with hematoxylin before dehydration and placement of the coverslips. The number of F4/80-positive cells per microscopic field was calculated per the similar intestine area.

#### Electron microscopy

Duodenum and jejunum samples were immediately fixed in 3% glutaraldehyde (Sigma) in PBS, post-fixed in osmium tetroxide, dehydrated and embedded in Epon 812. Ultrathin sections were cut (Reichert ultramicrotome), placed on mesh copper grids, counterstained with uranyl acetate and lead citrate and examined with a Hitachi 300 transmission electron microscope.

#### Measurement of parasympathetic nervous system activity

Mice were anesthetized with isoflurane to record the parasympathetic nerve activity of the vagus nerve at the level of the trachea. While the mice were kept on a heating blanket at 37°C in a Faraday cage, a first electrode was attached to the vagus nerve whereas a second one was implanted under the skin as a reference. A glucose solution (2 g/kg) was administered to the mice (still awake) through an intragastric catheter. After the intralipid or isocaloric infusion, the mice were anesthetized, and the vagus nerve activity was recorded 15 min before and 30 min after intragastric glucose administration. The signal was filtered between 0.1 and 1000 Hertz, with a 4 k/s sampling rate, and amplified by a BioAmp device (Phymep, Paris, France). At completion of the recording period 600 µg of acetylcholine were injected into the peritoneal cavity. This led to dramatically increased vagus nerve activity, thus validating the sensitivity of the experimental set up. Data were digitalized with PowerLab/4sp digitalizer (ADInstruments, Paris, France) and analyzed spectrally using a Spike Histogram (ADInstruments). Neuronal activity was normalized to reduce the variability resulting from differences in background activity.

#### Statistics

Results are presented as means ± SEM. The Student t-test was used to assess statistical significances between groups, except for ITT and GTT analysis, where Two-Way ANOVA with Bonferroni's post-test was applied. P<0.05 was considered statistically significant.
